# POST COVID-19 PANDEMIC EMPLOYMENT RECOVERY BY HEALTHCARE SECTOR IN US RACIAL MAJORITY-MINORITY COUNTIES

**DOI:** 10.1093/geroni/igad104.3530

**Published:** 2023-12-21

**Authors:** Lucille Xiang, Kiran Sreenivas

**Affiliations:** American Health Care Association / National Center for Assisted Living, Washington, District of Columbia, United States; American Health Care Association / National Center for Assisted Living, Washington, District of Columbia, United States

## Abstract

Health care sector employment trends following the COVID-19 pandemic have revealed a labor force participation gap. Racial/ethnic composition differences between counties may contribute to an uneven labor market recovery. This study’s objectives were to compare post-pandemic employment recovery between five health care sectors (ambulatory services (AS), home health care (HHC), nursing and residential care (NRC), assisted living (AL), nursing facilities/homes (NH)), and to examine whether health care sector recovery differences exist between racial majority-minority (MM) and non-racial MM counties in the USA. First Quarter 2020 and fourth Quarter 2022 data from the US Bureau of Labour Statistics Quarterly Census of Employment and Wages (QCEW) were used to calculate cumulative percentage change in health care sector employment. The Race Origin Variables from the latest 2021 data from the American Community Survey were used to identify racial MM counties. The sector with the greatest number of counties with positive employment recovery was the AS sector in 1562/3176 (49%) counties, and 252/468 (54%) MM counties. The NH sector had the lowest number of counties with positive employment recovery in 170/2818 (6%) counties, and 34/389 (9%) MM counties. This is the first analysis of post-pandemic health care sector employment recovery examining MM counties. Differences in county and state policies may account for varying degrees of recovery. Further research is necessary for the development of best practice guidelines and policies to improve post-pandemic health care employment recovery and the quality of nursing home care among older adults.

